# Prenatal Treatment of Mosaic Mice (Atp7a mo-ms) Mouse Model for Menkes Disease, with Copper Combined by Dimethyldithiocarbamate (DMDTC)

**DOI:** 10.1371/journal.pone.0040400

**Published:** 2012-07-18

**Authors:** Małgorzata Lenartowicz, Wojciech Krzeptowski, Paweł Koteja, Katarzyna Chrząścik, Lisbeth Birk Møller

**Affiliations:** 1 Department of Genetics and Evolution, Institute of Zoology, Jagiellonian University, Krakow, Poland; 2 Department of Cell Biology and Imaging, Institute of Zoology, Jagiellonian University, Krakow, Poland; 3 Institute of Environmental Sciences, Krakow, Poland; 4 Center of Applied Human Genetics, Kennedy Center, Glostrup, Denmark; Alexander Flemming Biomedical Sciences Research Center, Greece

## Abstract

Menkes disease is a fatal neurodegenerative disorder in infants caused by mutations in the gene *ATP7A* which encodes a copper (Cu) transporter. Defects in *ATP7A* lead to accumulated copper in the small intestine and kidneys and to copper deficiencies in the brain and the liver. The copper level in the kidney in postnatal copper-treated Menkes patients may reach toxic levels. The mouse model, *mosaic Atp7a* ***^mo-ms^*** recapitulates the Menkes phenotype and die about 15.75±1.5 days of age. In the present study we found that prenatal treatment of *mosaic* murine fetuses throughout gestation days 7, 11, 15 and 18 with a combination of CuCl_2_ (50 mg/kg) and dimethyldithiocarbamate (DMDTC) (280 mg/kg) leads to an increase in survival to about 76±25.3 days, whereas treatment with CuCl_2_ alone (50 mg/kg) only leads to survival for about 21 days ±5 days. These copper-DMDTC treated mutants showed an improved locomotor activity performance and a gain in body mass. In contrast to treatment with CuCl_2_ alone, a significant increase in the amount of copper was observed in the brain after prenatal copper-DMDTC treatment as well as a decrease in the amount of accumulated copper in the kidney, both leading towards a normalization of the copper level. Although copper-DMDTC prenatal treatment only leads to a small increase in the sub-normal copper concentration in the liver and to an increase of copper in the already overloaded small intestine, the combined results suggest that prenatal copper-DMDTC treatment also should be considered for humans.

## Introduction

Copper is an essential trace element for normal growth and development of all living organisms. Currently, more than thirty proteins are known in which copper serves as a cofactor due to its redox ability. In organisms, copper-dependent enzymes are essential to vital processes such as respiration, detoxification of free radicals, connective tissue formation and normal central nervous system function and development [1], [2], [3]. Copper maintains a delicate homeostasis at the cellular level and its metabolism is under genetic control. Menkes disease (MD) is an X-linked recessive disorder caused by mutations in the gene *ATP7A*, which encodes a copper-transporting P-type ATPase. The clinical features of MD are a consequence of a dysfunctional copper-distribution system and the malfunction of a large number of copper-requiring enzymes [4]. Classical MD is characterized by mental retardation, hypothermia, failure to thrive, convulsions, cutis laxa, motor dysfunction, spasticity and weakness of extremities, hypo-pigmentation, abnormal hair (kinky hair, pili torti) and decreased serum ceruloplasmin [5].

*Mottled* mice carry mutations in the homologous murine *Atp7a* gene. In mice mutations in the *Atp7a* gene also lead to disturbances in copper metabolism, and the severity of the phenotype is dependent on the specific *mottled* mutation in similarity to what is observed for patients with Menkes disease. The *11H* (*Mo^11H^*) mutation is embryonic lethal, the *macular* and *brindled* mutations are postnatal lethal (the mouse dies about 14–17 days after birth) and the blotchy mutation leads to a milder phenotype [6], [7], [8], [9]. The ATP7A protein is located in the trans-Golgi network and is involved in the ATP-dependent transport of copper across plasma or intracellular membranes. The ATP7A protein plays an important role in the release of dietary copper from the intestine to the body, including to the brain.

*Mosaic* mutation (*Atp mo-ms*) belongs to the group of *mottled* mutations which severely affect copper metabolism. The *mosaic* mutant males have severe symptoms similar to the *macular* and *brindled* mutant males, corresponding to classic Menkes disease. *Mosaic* mutant males die at about day 16. Analyses of the copper content in the organs of these mutants indicate that copper is accumulated in the small intestine and kidneys, while the brain, liver and heart suffer from copper deficiency [10], [11]. The initiation of copper supplement therapy during the neonatal period in the form of subcutaneous injections of CuCl_2_ improved the viability of the *mosaic* males and prolonged their survival [10], [12]. However, when combined with lipophilic chelators only small amounts of copper are necessary to obtain prolonged survival of *macular* males [9]. Dithiocarbamates, diethyldithiocarbamate (DEDTC) and dimethyldithiocarbamate (DMDTC) are chelators which form a lipofilic complex with Cu and facilitate copper transport across the cell membranes. Results of a previous study indicated that DMDTC was especially effective in the passage of copper across the blood brain barrier [9]. Therefore we examined, for the first time, the effect of a prenatal copper supplement combined with the DMDTC. We tested the effect on behaviour and survival and we measured the copper content in the brain, kidney, liver and small intestine of the mutant hemizygous males.

## Materials and Methods

### Animals

All mice used in these experiments came from the closed outbred colony (it means that the mice were maintained by mating unrelated mice using a numbers random table), at the Department of Genetics and Evolution of the Jagiellonian University. The experimental animals were obtained by mating A) normal wild-genotype females (+/+) with normal wild-type males (+/−), or B) heterozygous (ms/+) females with normal (+/−) males. The appearance of the vaginal plug counted as day 0. The mother mice were divided into four groups; I: Females treated with subcutaneous injections of 50 g CuCl_2_ (Sigma, 1 mg CuCl_2_/ml PBS), in the following designated “CuCl_2_”. II: Females treated with subcutaneous injections of 50 g CuCl_2_ (Sigma, 1 mgCuCl_2_/ml PBS) combined with intraperitoneal injections of 7 mg of sodium dimetyldithiocarbamate (DMDTC, Sigma, 70 mg/ml sterile H_2_O), in the following designated “CuCl_2_-DMDTC”. III: females treated with intraperitoneal injections of 7 mg of sodium dimetyldithiocarbamate (DMDTC, Sigma, 70 mg/ml sterile H_2_O), in the following designated “DMDTC”. IV: Untreated females, in the following designated” untreated” were used as a control group. Females were treated on day 7, 11, 15 and 18 of pregnancy. After delivery littermates stayed with the mother and suckled.

The 14-day-old hemizygous normal males (*+/−*) and mutant (*ms/−*) were differentiated on the basis of characteristic curly whiskers and the light fur of (*ms/−*) mice. Wild-type females and heterozygous females were distinguished on the basis of the characteristic light spots on the fur of heterozygous females.

The mice were housed at constant temperature (22°C) under an artificial light regime (12∶12 of day and night), and fed with the standard Murigran diet (Motycz, Poland).

The experiments were performed in accordance with Polish legal requirements under the licence of the Commission of Bioethics of the Jagiellonian University. The permission was obtained from the I Regional Ethics Committee on Animal Experimentation in Krakow, no. 47/2010 from 26. 05. 2010.

### Analysis of the Locomotors Activity

Individuals were placed on the arena (32×42 cm) and allowed to freely move for 3 minutes while being recorded by overhead camera (Sony DSC-H2). Video files were analysed with EthoVision XT-8 software (Noldus Information Technology; The Netherlands). Four parameters characterizing activity were computed: total distance moved (TotDist; cm), proportion of time spent moving (MoveTimeP), maximum speed of locomotion (Vmax; cm/sec), and the "Meander" index (average change of direction angle of movement per unit of distance moved; radians/cm). The latter parameter measures the degree of ambiguity or shakiness of the movement.

### Atomic Absorption Spectrometry

The level of copper in the organs of all experimental groups of mice was measured by atomic absorption spectrophotometry (AAS). The animals were sacrificed by cervical dislocation and their organs were excised and weighed. Samples were digested in 2 ml boiling Suprapur-grade nitric acid (Merck). After being cooled to room temperature, each sample was diluted to 10 ml with deionised water. Samples of reference material were prepared in a similar manner, where pure nitric acid was used as a blank sample and bovine liver (BRC No. 185, Prochem Gmbh, Germany) with a certified concentration of copper (189±4 mg/kg^−1^) was used for concentration determination.

### Statistical Analysis

Statistical comparisons of body mass, copper concentrations in tissues, litter size, sex ratio and mutant’s proportion were performed using the Student t-test, chi square test and Log-rank (Mantel-Cox) test. Locomotor activity was analyzed with two-way multivariate analysis of variance (MANOVA), with the four characteristics of locomotion (TotDist, MoveTimeP, Vmax, Meander) as dependent variables and Genotype (wild-type *versus* mutant), Treatment (treated with copper and DMDTC *versus* untreated animals) and Genotype×Treatment interaction as fixed factors. Significance of the effects of these factors on all the four activity traits considered simultaneously was tested with Wilks’ Lambda statistics, but univariate *F* tests were also performed separately for each of the traits. The values are expressed as mean ± SD, and p<0.05 was accepted as a level of significance.

## Results

### Description of Phenotype and Activity of the Investigated Animals

**Figure 1:**
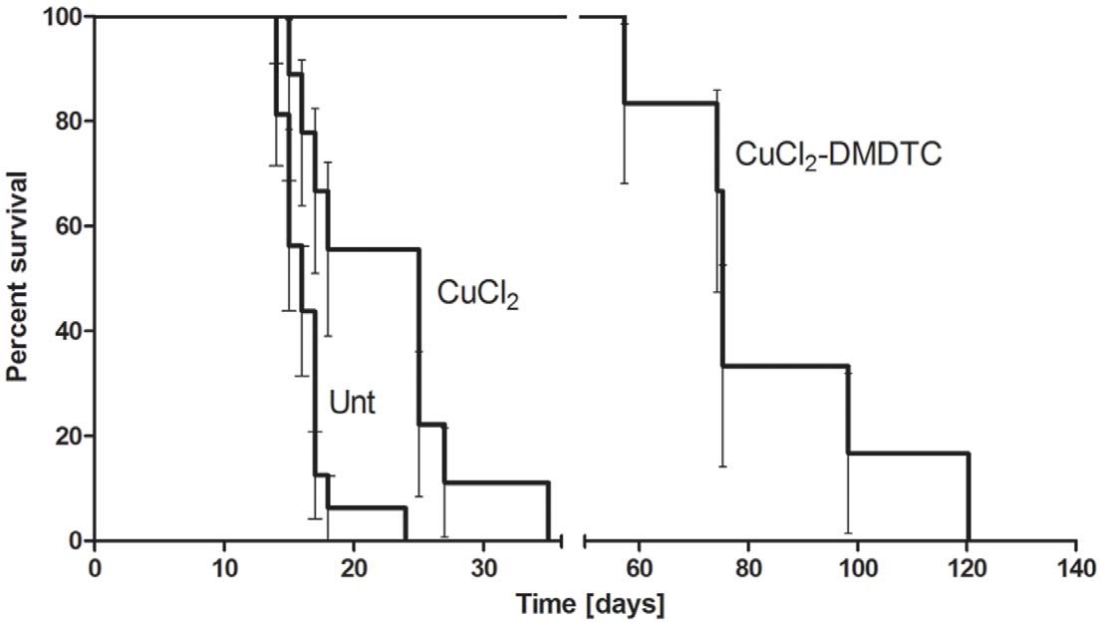
Kaplan-Meier plot of the effect of prenatal therapy on the survival of *mosaic* mutants. Data were analysed by log-rank Mantel – Cox test. Mutants born of CuCl_2_-DMDTC- treated mothers survive significantly longer (survival mean 79 days) than mutants born of untreated mothers (Unt.) (survival mean 16 days), P<0.0001, and mutants born of CuCl_2_-treated mothers (survival mean 21 days), P<0.001. Mutants born from CuCl_2_- treated mothers survive longer than mutants born of untreated mothers, P<0.01.

**Figure 2:**
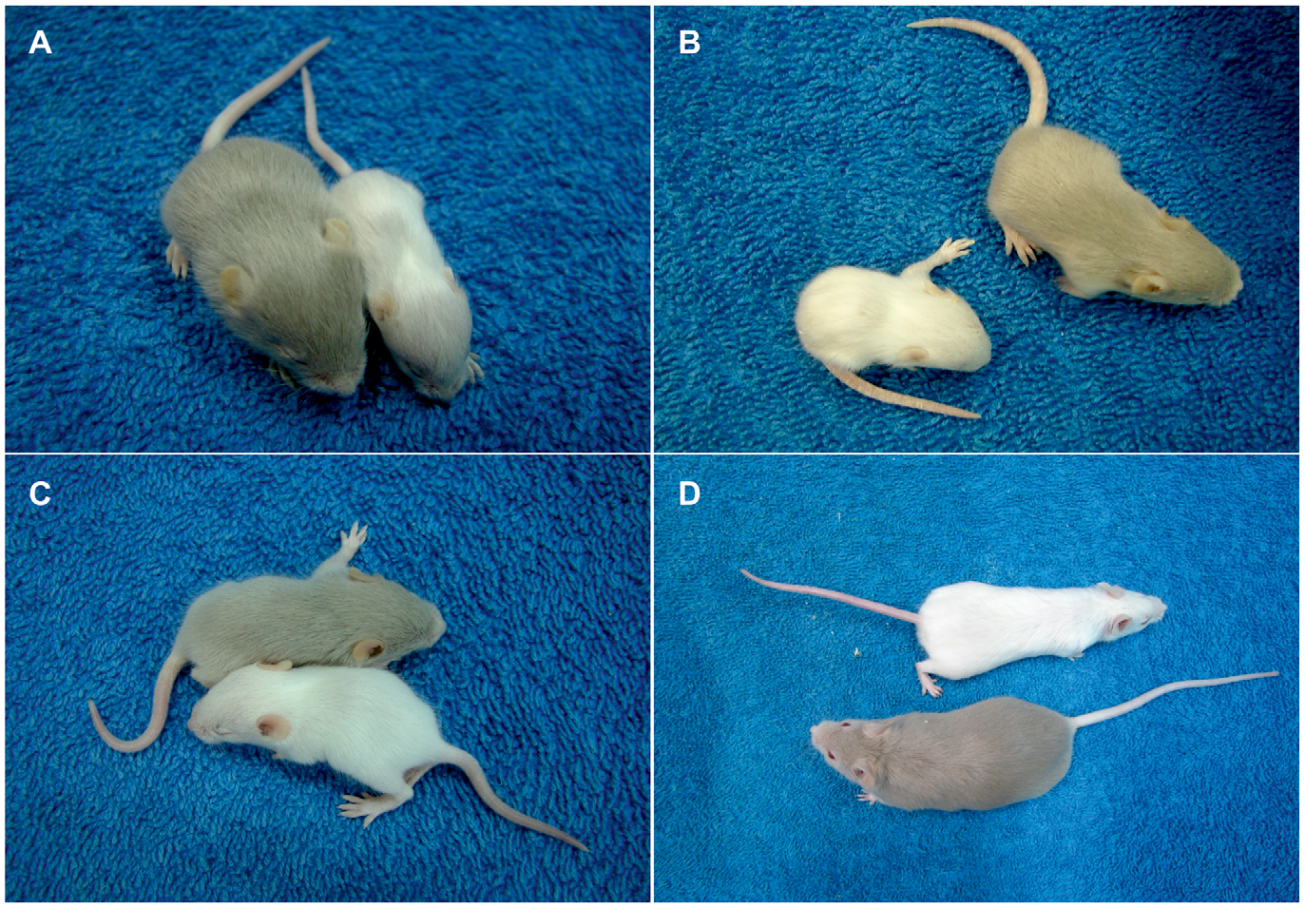
14-day-old mice; wild-genotype males (grey) and mosaic mutant males (white). The mice are born of: A) untreated heterozygous female. B) CuCl_2_-treated heterozygous female, C) CuCl_2_-DMDTC treated heterozygous female. The young mosaic mutant mice are smaller than the wild type mice. D) 8-week-old males, wild-genotype male (grey) and mosaic mutant male (white) born of a CuCl2-DMDTC treated heterozygous female.

We analyzed the effect of the prenatal treatment on the longevity and behavior of the mosaic mutants. Mutant progeny of untreated mothers die between 15–17 days (mean 15.75±1.5) after birth (Figure 1). 14-day-old mutants are depigmentated and suffer from neurological problems. Mutant mice are much smaller than their wild-type counterparts (Figure 2A and Video S12A). This result concurred with what has been published previously [10], [12]. *Mosaic* mutants derived from CuCl_2_-treated mothers survive the critical period and live longer than mutants with untreated mothers. Mutants also exhibit defect in pigmentation (Figure 2B and Video S2B) and they die at about 3 weeks of age (mean 21 days ±5; few survive for 27 days) (Figure 1). *Mosaic* males born of CuCl_2_-DMDTC-treated mothers survive for significantly longer (P<0.0001, data were analysed by log-rank Mantel – Cox test)- about two months (mean 76±25.3 days) - and the oldest of them, about 40%, live for more than 3 months (Figure 1). However the depigmentation was observed in both 14-day-old and older mutants, as shown on Figure 2C and 2D and in the Video S32C and S42D. Mice born of DMDTC treated mothers die before they were born (fetal resorption) or die few days after. Half of the mice died because the treated mothers died just after delivery. It was clear that treatment with DMDTC alone was toxic, so we terminated these experiments.

### The Effect of Prenatal Therapy on the Litter Size, Sex Ratio and Proportion of the Mutants

**Figure 3:**
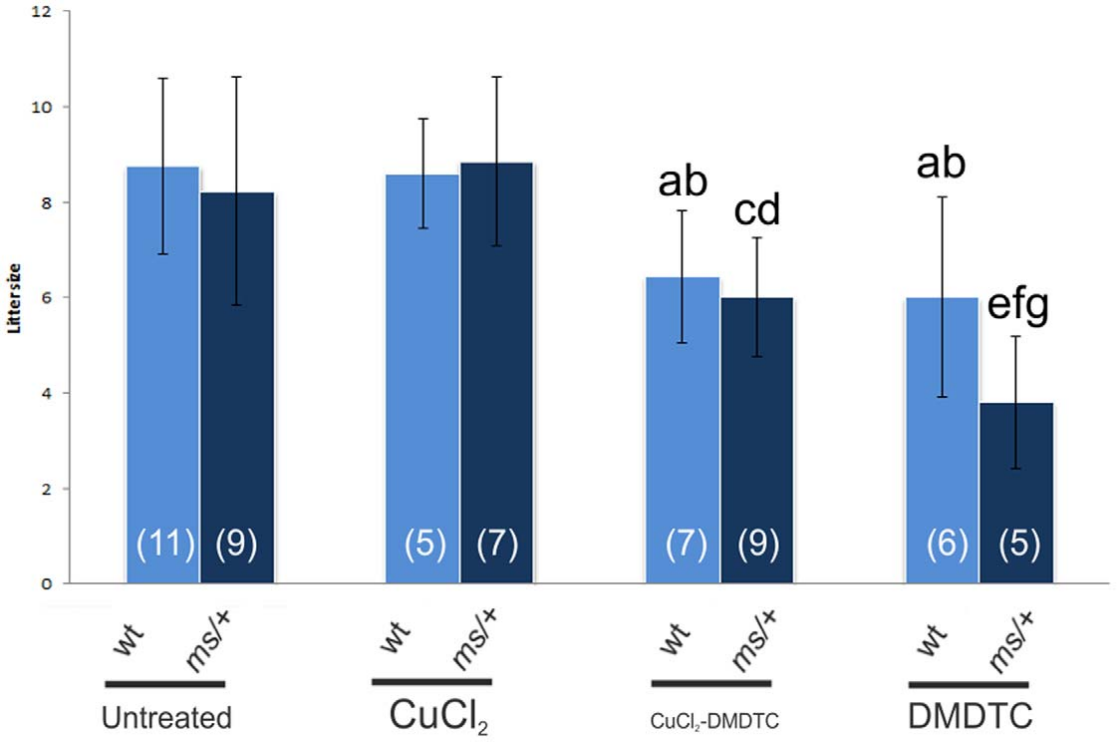
The effect of prenatal therapy on the litter size born from untreated, CuCl_2_-treated, CuCl_2_-DMDTC-treated or DMDTC-treated, wild type (wt) and heterozygous (ms/+) mothers. Significantly different from the litter size from ^(a)^ wild-type untreated mothers P<0.05; ^(b)^ from wild-type CuCl_2_-treated mothers P<0.05; ^(c)^ from untreated heterozygous mothers P<0.05; ^(d)^ from heterozygous CuCl_2_-treated mothers P<0.01; ^(e)^ from untreated heterozygous mothers P<0.01; ^(f)^ from heterozygous CuCl_2_-treated mothers P<0.001; ^(g)^ from heterozygous CuCl_2_-DMDTC- treated mothers P<0.05. The number of litters in each group is shown in brackets.

The litter size born from untreated and CuCl_2_-treated wild-type- and heterozygous mothers was similar but the litter size from the CuCl_2_-DMDTC-treated and the DMDTC treated female was decreased in comparison with untreated and CuCl_2_-treated mothers (Figure 3 and Table S1). Analysis of the data using the chi-square test indicated that sex ratio and proportion of the mutants in the progeny of heterozygous females was not significantly different in any of the investigated groups (Table S1).

### The Effect of Prenatal Therapy on the Body Weight of the 14-day-old Males

**Figure 4:**
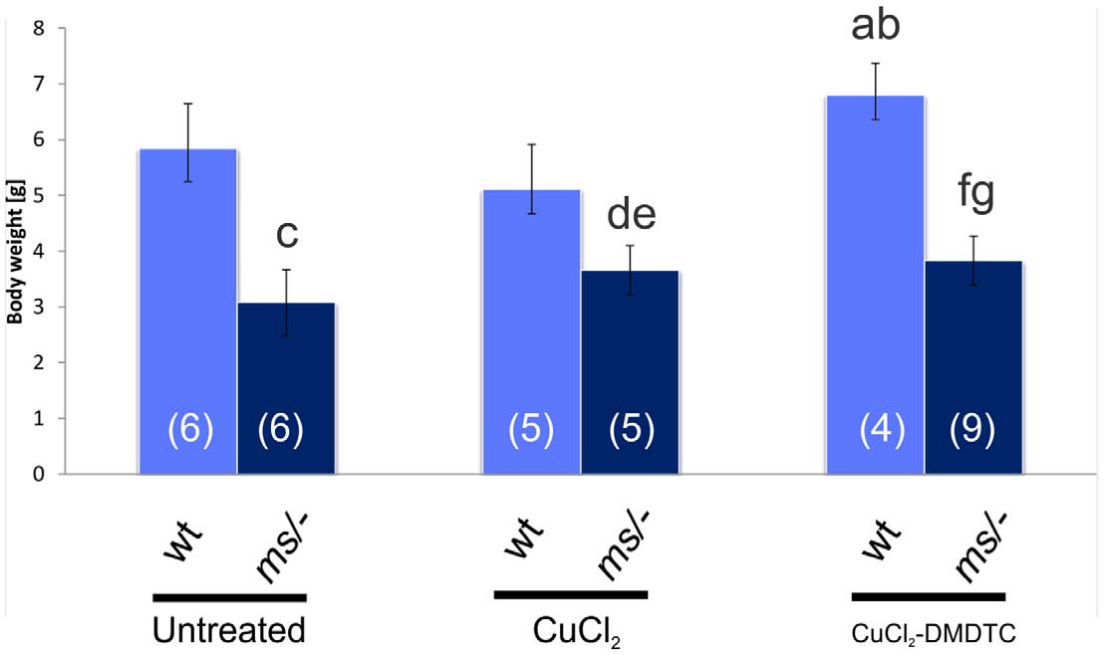
Body mass [g] of 14-day-old males (wild type (wt) or *mosaic* mutants (ms/−)) from untreated, CuCl_2_- or CuCl_2_-DMDTC-treated heterozygous mothers (ms/+); ^(a)^ Significantly different from untreated control wild-type males P<0.05; ^(b)^ Significantly different from CuCl_2_-treated wild-type males P<0.01; ^(c)^ Significantly different from untreated control wild-type males P<0.0000; ^(d)^ Significantly different from CuCl_2_-treated wild-type males P<0.001; ^(e)^ Significantly different from untreated mosaic males P<0.05; ^(f)^ Significantly different from CuCl_2_-DMDTC-treated wild-type males P<0.0000; ^(g)^ Significantly different from untreated mosaic males P<0.05. There is no difference between prenatal CuCl_2_-treated and prenatal CuCl_2_-DMDTC treated mosaic males. The number of mice in each group is shown in brackets.

The body mass of the prenatally CuCl_2_-treated as well as prenatally Cu-DMDTC-treated mutants was higher (P<0.05) than that of the untreated *mosaic* males (Figure 4, Table S2). However, it was still lower than the body mass of the wild-genotype males. In the group of wild-genotype males, the body mass of the prenatally CuCl_2_-DMDTC-treated mice was higher than the untreated (P<0.05) and the prenatally CuCl_2_ treated mice (P<0.01) (Figure 4, Table S2).

The CuCl_2_-DMDTC- treated mutant mice was smaller than the wild-type mice (Figure 4). Also 6-week old and 2-month old prenatally CuCl_2_-DMDTC-treated mosaic males were smaller than the wild-type males (Figure 2D and Video S42D). For older mice the differences in the size between CuCl_2_-DMDTC-treated and wild type individuals disappeared (not shown). Only few hemizygous males survive that long.

### Description of Locomotor Activity of the Investigated Animals

**Figure 5:**
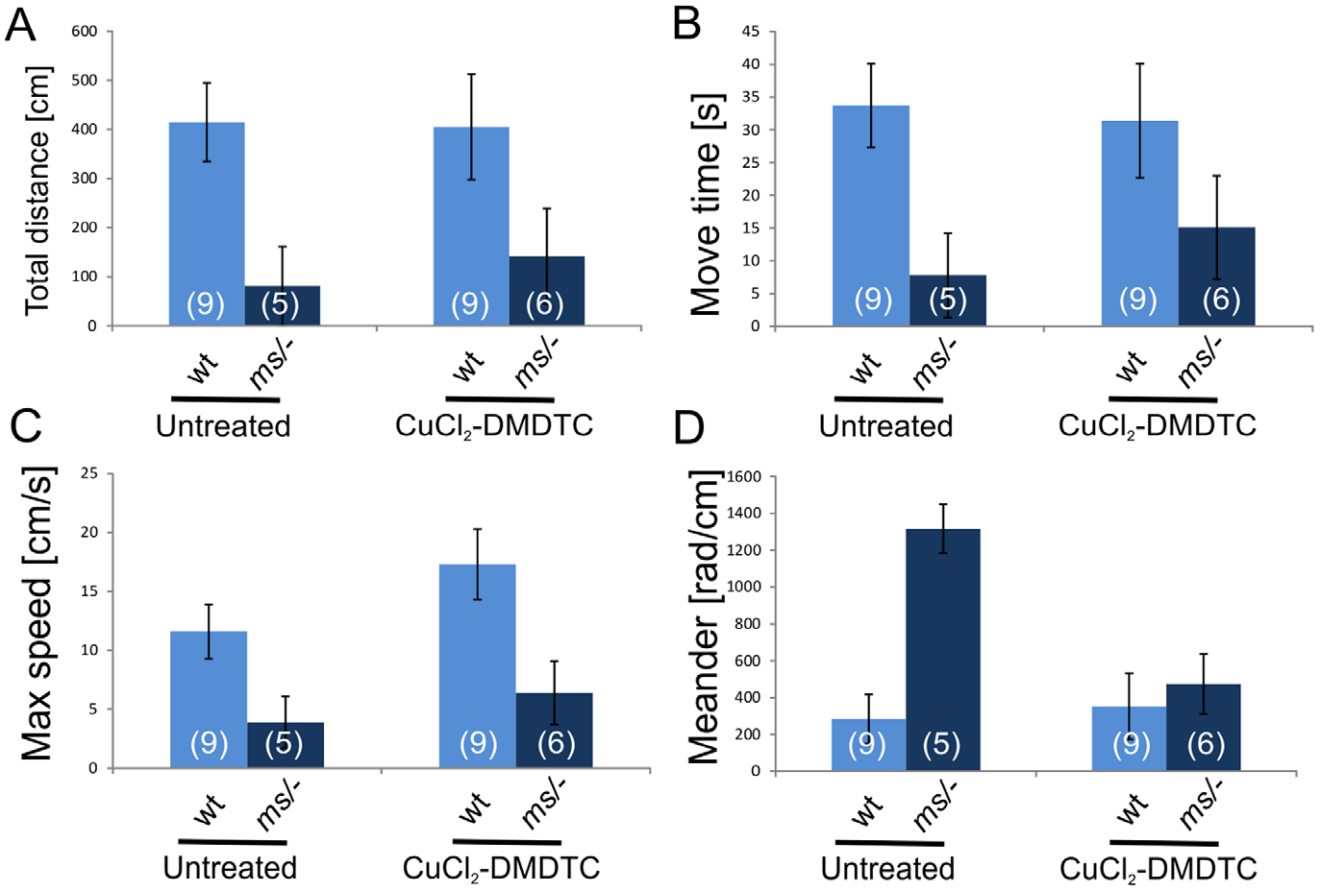
Analysis of the activity and movement parameters of 14-day-old mosaic mutant (ms/−) and wild-genotype males (wt) born of untreated, CuCl_2_- or CuCl_2_-DMDTC-treated heterozygous mothers. A) TotDist - total distance moved (cm) by center of body; B) MoveTimeP - proportion of time spend moving; C) Vmax - maximum running speed; D) Meander – absolute change of angle of movement per unit distance (direction of change not distinguished; high values indicate wavering movement). See Table S3 for results of MANOVA.

Mutants born of untreated mothers, exhibit problems with locomotion (Video S12A), they suffer from ataxia and often from a paralysis of the hind limbs. Locomotor activity of the 14-day-old wild-genotype and mutant males, differed with respect to all four traits analyzed (Wilks’ Lambda  = 0.569, F_4,22_ = 4.16, P = 0.012; Figure 5 and Table S3). Compared to wild-type males, the mutants had a lower proportion of time spend moving (MoveTimeP; P = 0.009), total distance moved (TotDist P = 0.003), and maximum running speed (Vmax; P = 0.001), but a higher value of the Meander index (P = 0.001), which indicated a more wavering movement of the mutants (Figure 5 and Table S3). Mutants derived from CuCl_2_-treated mothers survived longer but they also exhibit neurological problems such as tremor and convulsions, in most cases, these 14-day-old mutants, exhibit neurological problems similar to those seen in mutants with untreated mothers (Videos S12A and S2B). They had ataxia and developed a paralysis of the hind limbs. They are also less active than their wild-type brothers of the same age. (Video S2B). Mutant males born of CuCl_2_-DMDTC-treated mothers were, from the age of 14 days onwards, just more active than untreated *mosaic* males, however the activity was still lower than in wild-genotype males (Figure 5 and Table S3). The multivariate test showed significant interaction between the Genotype and Treatment effects (Wilks’ Lambda = 0.608, F_22,4_ = 3.55, P = 0.022), which means (in combination with results for main effects) that the treatment significantly improved locomotor performance of mutant males, but did not markedly affect locomotion of wild-type males. This is not surprising, because it was not expected that it should affect wild-type males. Prenatal treatment with CuCl_2_ and chelator improved to some extent all aspects of the locomotor performance in mutant males, but univariate analyses showed that the effect was significant only for the Meander index (Figure 5 and Table S3). Thus, the treatment improved primarily "stability" of locomotion (low meander value) and to a smaller extent its duration or intensity (Figure 5 and Table S3). According to our observation CuCl_2_ and chelator treated 14 days old mutants moved similar to wild-genotype males and did not have any obvious neurological problems (Video S32C). Tremor, convulsions or paralysis of the hind limbs were not observed in any of these mice. *Mosaic* males (about 60 days old) born of CuCl_2_-DMDTC-treated mothers are shown on Video S42D. They were caged with the females, but none of the mutant males were fertile.

### Copper Concentration in the Organs of the Progeny of Wild-type Mothers

Copper content in the organs of the 14-day-old wild-type male mice with wild-type mothers were measured. The copper concentration in the liver of the progeny of CuCl_2_ and CuCl_2_-DMDTC-treated wild-type mothers, was significantly higher (P<0.001 and P<0.05 respectively) than the concentration in the progeny of untreated females. The copper concentration in the other organs was similar in all experimental groups of mice. Results are summarized in Figure S1 and Table S4.

### Copper Concentration in the Organs of the Progeny of Heterozygous Mothers

**Figure 6:**
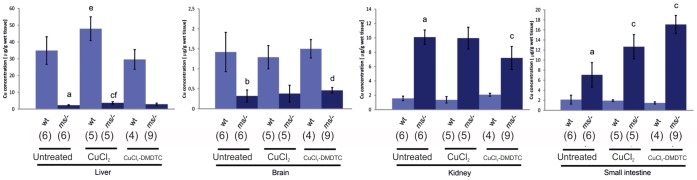
Copper concentration (g/g wet tissue) in the organs of the 14-day-old progeny of untreated, CuCl_2_- or CuCl_2_-DMDTC treated heterozygous mothers. ^(a)^ Significantly different from wild-type animals P<0.01; ^(b)^ Significantly different from wild-type animals P<0.000; ^(c)^ Significantly different from untreated mutant animals P<0.01; ^(d)^ Significantly different from untreated mutants P<0.05; ^(e)^ Significantly different from untreated mutants P<0.05; ^(f)^ Significantly different from wild-type animals P<0.001. The number of mice in each group is shown in brackets.

Among the littermates obtained from heterozygous mothers crossed with wild-type males there are normal wild-type males and females, mutant males and heterozygous females. We analysed the copper content in the organs of the 14-day-old males. Analysis of the copper concentration in the organs of progeny in the group with untreated mothers showed that the copper content in the liver and brain of mutant males is significantly lower compared to that in control wild-type males, whereas the copper content is increased in the small intestine and kidney (Figure 6 and Table S5). This concurs with previously published data [10].

After administering CuCl_2_-injections to pregnant females, their mutant male off-spring had higher liver copper-concentrations than did mutants from untreated females (P<0.01). However, the copper concentration in the liver in the mutant males was still significantly lower than in the control wild-type males (P<0.001). An increased copper level was also found in the small intestine of mutant males from CuCl_2_-treated mothers, as compared to the control mutants (P<0.01).

The mutant males born of CuCl_2_-DMDTC-treated mothers had an increased copper concentration in the brain and in the intestine as compared to the control mutant males (Figure 6 and Table S6). We also found a small increase in the copper concentration (non significant) in the liver of the mutant males born of the CuCl_2_-DMDTC-treated mothers. Surprisingly, the copper content in the kidneys of the prenatally CuCl_2_-DMDTC- treated mutants wereas significantly lower than in the group of prenatally CuCl_2_-treated and -untreated mutants P<0.01, but it was still higher than in wild-type males.

Wild type male progeny of the CuCl_2_-treated heterozygous females exhibited an increased copper concentration in the liver. There was no difference in the copper concentrations in any of the other organs of wild-type progeny, irrespective of the treatment (Figure 6 and Table S5).

### Copper Concentration in the Organs of the Wild-type and Heterozygous Adult Females

We also analysed the copper concentration in the livers and kidneys of the treated mothers. Copper concentrations in the liver and in the kidney were similar in all groups of wild-type and heterozygous females respectively (Table S6). However the kidney copper concentrations in the heterozygous mothers were 5 fold higher than in the wild-type females.

## Discussion

In the present study we focused on the effects of the prenatal treatment. Our previous results indicated that postnatal CuCl_2_ therapy of *mosaic* males from the second day of life improved the viability and survival substantial [12], [13]. Here we present the first description of the effect of combined (CuCl_2_ and DMDTC) prenatal treatment of mosaic mice from gestation day 7 to delivery. We tested the effect on, survival, activity and copper content in the brain, kidney, liver and small intestine of the mutant hemizygous *mosaic* males. *Mosaic* mice with altered copper metabolism appear to be a good model for the investigation of possible therapeutic effects of copper administration to patients with Menkes disease. Two-week-old *mosaic* mutants often exhibit neurological symptoms very similar to those seen in patients with Menkes disease, such as ataxia, tremor and convulsions [11].

We found that prenatal treatment with a combination of CuCl_2_ (50 mg/kg) and DMDTC (280 mg/kg) leads to a substantial increase in survival from 2 weeks to 11 weeks. In contrast, we found that treatment with CuCl_2_ alone (50 mg/kg) only leads to an increase in survival from 2 weeks to about 3 weeks. The seemingly good health of the prenatally CuCl_2_-DMDTC treated mutants is also confirmed by the increase in body size and mass. The two–month-old CuCl_2_-DMDTC- prenatally treated hemizygous male had almost the same size as a wild-type male (Figure 2D). Increased body weight was also found in the group of the CuCl_2_-DMDTC- prenatally treated wild-genotype males (Figure 4). Previously we reported that postnatal CuCl_2_ injection lead to significantly increasing in the body weight in wild-genotype males [11], [13]. A similar effect was observed in the pigs in which high dietary copper promotes growth and body weight and improved feed intake. It was explained by the fact that copper up-regulates the expression of appetite- regulated genes in the brain [14], [15]. The present data indicate that only prenatal treatment with CuCl_2_-DMDTC leads to an increase in the body mass in wild-type as well as mutant mice, whereas CuCl_2_ alone only leads to an increase in body mass of the mutant mice. This difference correlates with the copper concentration in the brain (Figure 6). Also the difference in treatment-strategy; prenatal versus postnatal and treatment route might however influence the effect. These experiments suggest that the lipophilic copper-complex was able to pass through the blood brain barrier (BBB). Inside the brain the copper is probably released as a result of reversibility of the DMDTC binding.

Positive effects of postnatal copper therapy have already been demonstrated in humans and mice, but only if the treatment is started within a few days after birth. Copper treatment administered before the 7^th^ day of postnatal life improved the viability of mottled mutant males; *mosaic* [11], [13], *brindled* [16] and *macular* mice [9], [17]. Tanaka and co-workers found that treatment of *macular* mice on the 7^th^ day with a subcutaneous injection of high amount of copper alone (10 mg/kg) or low amount of copper (1 mg/kg) combined with an intraperitoneal injection of DMDTC (100 mg/kg) leads to 100% survival for at least 7 weeks. Mice treated with low amount of copper (1 mg/kg) alone, all died after 2–3 weeks. In humans, among 12 newborns who were treated early with copper, survival at a median follow-up of 4.6 years was 92%, as compared with 13% at a median follow up of 1.8 years for a control group [18].

However the prenatally CuCl_2_-DMDTC-treated mutant male mice, although very active, die suddenly after about 2 months. Based on previous publications [9], [32], it is likely that a combination of prenatal and postnatal treatments leads to longer survival.

It has been suggested that the ATP7A protein plays an important role in maintaining copper homeostasis in the CNS. It is highly expressed in ependymal cells of the chorid plexus - a structure that regulates the concentration of the different molecules in the cerebrospinal fluid. It has also been proposed that ATP7A is involved in copper transport across the blood-brain barrier [1], [19], [20], [21]. The brain is especially sensitive to copper deficiency due to copper-dependent enzymes, such as cytochrome c oxidase, peptidylglicine alfa-amidating monooxygenase (PAM), dopamine-beta-monooxygenase and Cu,Zn-superoxide dismutase (SOD) [1], [22], [23]. The severe symptoms combined with mental retardation observed in patients with Menkes disease and in mouse models are probably due to the low copper concentration in the brain [1], [9], [12], [16], [24]. We also found a significantly lower concentration of copper in the brain of the mosaic mutant males than in the wild-type males. Thus, the most important objective when treating neurological degeneration associated with Menkes disease seems to be the delivery of copper across the blood–brain barrier and into the brain. For this purpose we combined the copper treatment with DMDTC which forms a lipophilic complex with copper, and therefore most likely facilitates the transport of copper by-pass the blood-brain barrier [9]. We found that prenatal treatment with a combination of CuCl_2_ and DMDTD, but not with CuCl_2_ alone (or only very slightly) leads to increased copper content in the brain (measured after 14 days). Chelators have a positive effect on the transport of copper to the brain. Postnatal treatment on day 7 with subcutaneous injections of CuCl_2_ combined with an intraperitoneal injection of DEDTC (sodium diethyldithiocarbamate, 50 mg/kg) increase the copper concentration significantly in the cerebellum [9], [25], although it does not reach the normal value. When the chelator DEDTC was replaced by DMDTC (50 mg/kg), the copper concentration in the brain (determined after 2 days) had increased to a normal value [9]. All previous investigations found that the administration of CuCl_2_ alone had no effect on the copper concentration in the brain, except for when very high concentrations of copper (10 mg/kg) were used [9]. The combined results - with respect to increasing the copper level in the brain - suggest that CuCl_2_-DMDTC therapy is more effective than CuCl_2_ therapy alone. In the present study we found that untreated mutant males have impaired all four aspects of locomotor performance analyzed in this work, and that the treatment with CuCl_2_ and chelator improved all aspects of the locomotor performance in mutant males, especially the stability of the movement. We suggest that the observed better motor and neurological behaviour in the prenatally treated mice can be subscribed to the increased copper concentration in the brain. It is also important that the treatment with CuCl_2_ and chelator did not affect locomotor performance of wild-genotype males. In our experiments we also analysed the locomotor performance of wild-genotype and heterozygous females (data not shown) but we did not found any differences between this two genotypes and no effect of the prenatal treatment could be observed.

Copper plays an essential role during development in mammals. Copper deficiency during development results in anatomical abnormalities in affected fetuses and/or embryonic death [26]. During pregnancy, the concentration of copper in the maternal serum rises [25], [27] and copper is transported from the maternal to the fetal circulation via the placenta. This process is mediated by ATP7A and ATP7B [2], [26]. In mammalian fetal life, copper is transported via the bloodstream from the placenta to the liver, where it is stored in the Cu-metallothionein complex [28], [29], [30]. The copper content in the fetal liver is higher than in adult liver and reaches a maximum concentration on day 16 of pregnancy [27]. In concurrence with previously published results, we showed that CuCl_2_ administration to the female during pregnancy leads to an increase in the copper concentration in the liver of the fetus [25], [27], [30], [31]. We showed that the copper concentration in the 14-day-old fetus had increased. This indicates that the effect of prenatal therapy persists to the second week of postnatal life. We also found a slightly higher copper content in the liver of hemizygous fetus after prenatal treatment with a combination of CuCl_2_ and DMDTC. In summary, the results suggest that prenatal treatment with CuCl_2_ is more effective than prenatal treatment with a combination of CuCl_2_ and DMDTC with regard to normalizing the copper level in the liver.

In the small intestine, dietary copper is transferred from the enterocytes into the bloodstream via the ATP7A protein. In Menkes patients and *mottled* mutant mice, ATP7A is defective and copper export from enterocytes is greatly impaired, resulting in copper accumulation in the intestinal cells and a restricted copper supply to other tissues [1]. However, our results indicate that the copper concentration in the intestinal cells in the mutant mice increased as a result of prenatal treatment, even though the copper was not given orally, but through the umbilical vein. We showed that the copper content in the small intestine of the CuCl_2_-treated - and to a higher degree - of the combined CuCl_2_ and DMDTC prenatally treated mutant mice was significantly higher when compared to untreated mosaic males. Kodama and co-workers showed that subcutaneous injections of CuCl_2_ (4 µg) and intraperitoneal injections of DMDTC (0.2 mg/g) lead to an increase in copper concentration in the small intestine of the macular mutants, even though this therapy was started on postnatal day 28 [32]. The combined data suggest that copper, and especially CuCl_2_-DMDTC (and CuCl_2_-DMDTC) complex that is transported through the blood is trapped in the enterocyte cells.

The kidney is also affected by disturbances in the copper metabolism in both patients with Menkes disease and in *mottled* mice, leading to pathological changes [4], [11], [33], [34]. The uptake of copper from urine is normal, but the mechanism of its reabsorption from the cell into the circulatory system is affected due to low ATP7A protein activity and therefore traps copper in the epithelial cells of the proximal tubules [35], [36], [37]. Our previous results showed that the copper accumulation process in the kidney is strongly enhanced during postnatal life, as the copper content in the kidney of 14-day-old mutants increased 4 fold, whereas the copper content in 1-day-old mosaic males only increased 2 fold compared to wild-type counterparts [10], [38]. Furthermore, postnatal CuCl_2_ treatment leads to a 10 fold increase in copper content as compared to that in untreated mutants [10]. But our current results showed that the copper content in the kidney of the mutants treated prenatally with CuCl_2_ is very similar to the copper content in the kidney of untreated mutants. Moreover, the copper concentration in the kidney of the mutants treated prenatally with a combination of CuCl_2_ and DMDTC was significantly lower than in the untreated mutants.

This could indicate that prenatal treatment with CuCl_2_ combined with DMDTC is also effective in decreasing the copper level in the kidney of patients with Menkes disease. Kidney damage brought on by copper accumulation is particularly interesting, as copper therapy and subsequent kidney damage is observed in patients with Menkes disease [33], [34], [39].

Our results also show that the copper level in the liver and kidney of the heterozygous CuCl_2_-DMDTC treated mothers had not changed, indicating that prenatal therapy does not have a toxic effect on the mother (Table S6). However the numbers of offspring obtained from CuCl_2_-DMDTC treated females was smaller than from untreated and CuCl_2_-treated females (Figure 3). Moreover in the present study we have big problems obtaining progeny from the females treated only with DMDTC. From the DMDTC treated heterozygous females we obtained only a few litters and the size of the litters was also very small. It may indicate that DMDTC treatment (in the absence of copper) has a toxic effect on prenatal development in mice. It has previously been shown that dithiocarbamates have a toxic effect on zebra fish development [40]. Before prenatal therapy of human Menkes disease patients is a real possibility different DMDTC related chelators should be evaluated for the ability to transport copper through the BBB combined with the toxicity. A less toxic chelator is to be preferred.

In conclusion, the results presented here indicate that when a supplement of CuCl_2_ combined with DMDTC is given to heterozygous mothers during pregnancy, the most serious problems posed by Menkes disease is probably improved. It increases the amount of copper in the brain and decreases the amount of copper in the kidney, and most importantly, it prolonged the survival of the progeny and the movement stability. Although toxicity considerations may prohibit long-term use of such agents in humans, the principle that lipid-soluble complexes can enhance copper transport across cellular membranes deserves further attention and should be considered in treatment of individuals with Menkes kinky hair disease. Experiment investigating the effect of CuCl_2_ versus CuCl_2_-DMDTC therapy on mice treated prenatally with CuCl_2_-DMDTC is ongoing in our laboratory.

## Supporting Information

Figure S1 **Copper concentration (g/g wet tissue) in the organs of the 14-day-old wild-type progeny of untreated, CuCl_2_- or CuCl_2_ -DMDTC treated wild-type mothers.** ^(a)^ Significantly different from untreated animals P<0.05; ^(b)^ Significantly different from untreated animals P<0.001. The number of mice in each group is shown in brackets.
(TIF)

Table S1 **Total number of progeny and sex ratio of the progeny obtained from wild type and heterozygous females.**
(RTF)

Table S2 **Body mass [g] of the 14-day-old males with heterozygous mothers.** Significantly different from untreated control wild-genotype males P<0.05; ^(b)^ Significantly different from Cu-treated wild-genotype males P<0.01; ^(c)^ Significantly different from untreated control wild-genotype males P<0.0000; ^(d)^ Significantly different from Cu-treated wild-genotype males P<0.001; ^(e)^ Significantly different from untreated mutant males P<0.05; ^(f)^ Significantly different from Cu-DMDTC-treated wild-genotype males P<0.0000; ^(g)^ Significantly different from untreated mutant males P<0.05.
(RTF)

Table S3 **Results of multivariate analysis of variance of four activity traits in 14-day old males (performed with GLM procedure of SYSTAT 11 statistical package; see Methods for description of the model).**
(RTF)

Table S4 **Cu concentration (g/g wet tissue) in the organs of the 14-day-old wild-type progeny of wild-type mothers.** ^(a)^ Significantly different from untreated animals P<0.05; ^(b)^ Significantly different from untreated animals P<0.001.
(DOCX)

Table S5 **Cu concentration (g/g wet tissue) in the organs of the 14-day-old progeny of the heterozygous mothers.** ^(a)^ Significantly different from wild-type animals P<0.01; ^(b)^ Significantly different from wild-type animals P<0.000; ^(c)^ Significantly different from untreated mutant animals P<0.01; ^(d)^ Significantly different from untreated mutants P<0.05; ^(e)^ Significantly different from untreated mutants P<0.05; ^(f)^ Significantly different from wild-type animals P<0.001.
(RTF)

Table S6 **Cu concentration (g/g wet tissue) in the organs of the wild-type and heterozygous mothers.**
(RTF)

Video S1 **14-day-old mutants and control mice from untreated mothers.**
(AVI)

Video S2 **14-day-old mutants and control mice from CuCl_2_-treated mothers.**
(AVI)

Video S3 **14-day-old mutants and control mice from CuCl_2_-DMDTC treated mothers.**
(AVI)

Video S4 **Approximately 60 days old mutants and control mice from CuCl_2_-DMDTC treated mothers.**
(AVI)
