# Supplementary figures and images for: Prenatal Treatment of Mosaic Mice (Atp7a mo-ms) Mouse Model for Menkes Disease, with Copper Combined by Dimethyldithiocarbamate (DMDTC)
Source: PLoS One. 2012 Jul 18;7(7):e40400. doi: 10.1371/journal.pone.0040400 (PMC3399861; doi:10.1371/journal.pone.0040400)

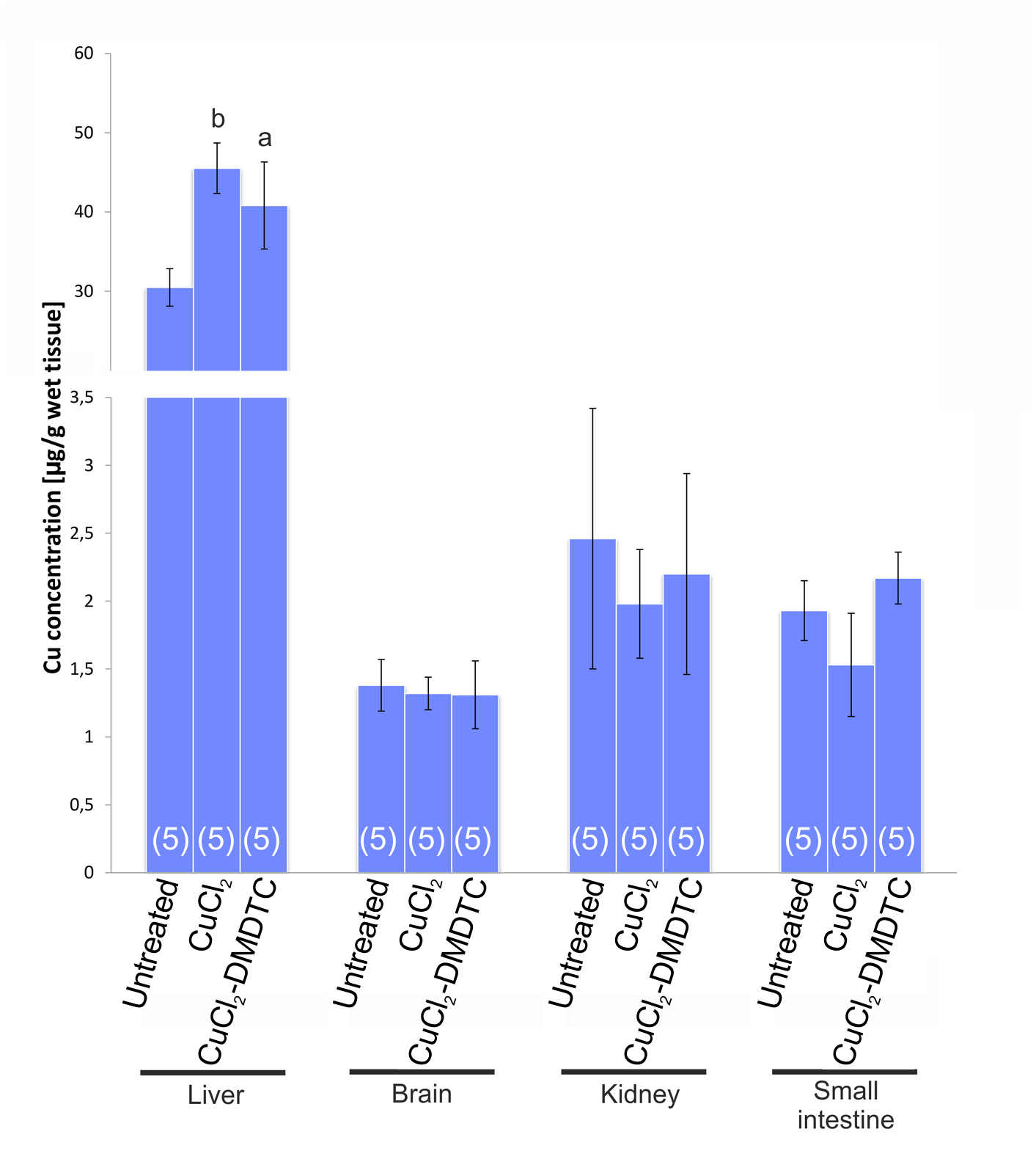

Supplement: Figure S1 — Copper concentration (g/g wet tissue) in the organs of the 14-day-old wild-type progeny of untreated, CuCl2- or CuCl2 -DMDTC treated wild-type mothers. (a) Significantly different from untreated animals P<0.05; (b) Significantly different from untreated animals P<0.001. The number of mice in each group is shown in brackets. (TIF) [file pone.0040400.s001.tif]
