# Supplementary material for: Prenatal Treatment of Mosaic Mice (Atp7a mo-ms) Mouse Model for Menkes Disease, with Copper Combined by Dimethyldithiocarbamate (DMDTC)
Source: PLoS One. 2012 Jul 18;7(7):e40400. doi: 10.1371/journal.pone.0040400 (PMC3399861; doi:10.1371/journal.pone.0040400)
Supplement: Table S1 — Total number of progeny and sex ratio of the progeny obtained from wild type and heterozygous females. (RTF) [file pone.0040400.s002.rtf]

Table S2. 

	Body weight 	
Heterozygous females x wild-type males	Intact 	CuCl2-treated	CuCl2-DMDTC-treated	
Wild-type males 
Mutant males 	5,84  0.80
3.08  0.59c	5,11  0.80
3.66  0.44de	6.80  0.57ab
3.83  0.44fg	
