# Supplementary material for: Prenatal Treatment of Mosaic Mice (Atp7a mo-ms) Mouse Model for Menkes Disease, with Copper Combined by Dimethyldithiocarbamate (DMDTC)
Source: PLoS One. 2012 Jul 18;7(7):e40400. doi: 10.1371/journal.pone.0040400 (PMC3399861; doi:10.1371/journal.pone.0040400)
Supplement: Table S3 — Results of multivariate analysis of variance of four activity traits in 14-day old males (performed with GLM procedure of SYSTAT 11 statistical package; see Methods for description of the model). (RTF) [file pone.0040400.s004.rtf]

Table S3. 
A) Least square means with standard errors for four combinations of two main factors (Genotype and Treatment): 

Factors	N	Parameters of activity	
Treatment	Parents' genotype		TotDist
mean ±  SE	MoveTimeP
mean ±  SE	Vmax
mean ±  SE	Meander
mean ±  SE	
Intact mothers	Wild type
Mutant	9
5	415 ±  80
81 ± 107	33.7 ± 6.4
7.8 ± 8.7	11.6 ± 2.3
3.9 ± 3.0	284 ± 133
1317 ± 179	
CuCl2-DMDTC treated mothers	Wild type
Mutant	9
6	405 ± 80
142 ± 97	31.4 ± 6.4
15.1 ± 7.9	17.3 ± 2.2
6.4 ± 2.7	353 ± 133
474 ± 163	

B) Tests for the effect of Genotype (wild-type versus mutant):
Univariate F Tests
Source             SS    df       MS           F              P
TOTDIST        607660     1   607660         10.595          0.003
  Error       1433865    25    57355
MOVETIMEP        3019     1     3019          8.036          0.009
  Error          9391    25      376
VMAX              606     1      606         13.162          0.001
  Error          1151    25       46
MEANDER       2263042     1  2263042         14.117          0.001
  Error       4007590    25   160304
 
Multivariate Test Statistics
Statistic             Value  F-Statistic       df             P
Wilks' Lambda         0.569    4.159          4, 22          0.012
---------------------------------------------------------------------------------------------------------------------
C) Tests for the effect of Treatment (untreated versus Cu-DMDTC treated):
Univariate F Tests
Source             SS    df       MS           F              P
TOTDIST          4575     1     4575          0.080          0.780
  Error       1433865    25    57355
MOVETIMEP          44     1       44          0.116          0.736
  Error          9394    25      376
VMAX              107     1      107          2.320          0.140
  Error          1151    25       46
MEANDER       1014036     1  1014036          6.326          0.019
  Error       4007590    25   160304
 
Multivariate Test Statistics
Statistic             Value  F-Statistic       df             P
Wilks' Lambda         0.715    2.197          4, 22          0.103
---------------------------------------------------------------------------------------------------------------------
D) Tests for the effect of interaction between Genotype and Treatment factors:
Univariate F Tests
Source            SS     df       MS           F              P
TOTDIST          8606     1     8606          0.150          0.702
  Error       1433865    25    57355
MOVETIMEP         154     1      154          0.411          0.527
  Error          9391    25      376
VMAX               14     1       14          0.312          0.581
  Error          1151    25       46
MEANDER       1410869     1  1410869          8.801          0.007
  Error       4007590    25   160304
 
Multivariate Test Statistics
Statistic             Value  F-Statistic       df             P
Wilks' Lambda         0.608    3.547          4, 22          0.022
---------------------------------------------------------------------------------------------------------------------
