# Supplementary material for: Prenatal Treatment of Mosaic Mice (Atp7a mo-ms) Mouse Model for Menkes Disease, with Copper Combined by Dimethyldithiocarbamate (DMDTC)
Source: PLoS One. 2012 Jul 18;7(7):e40400. doi: 10.1371/journal.pone.0040400 (PMC3399861; doi:10.1371/journal.pone.0040400)
Supplement: Table S4 — Cu concentration (g/g wet tissue) in the organs of the 14-day-old wild-type progeny of wild-type mothers. (a) Significantly different from untreated animals P<0.05; (b) Significantly different from untreated animals P<0.001. (DOCX) [file pone.0040400.s005.docx]

Table S4.

| Parents’ genotype | Cu concentration (μg/g wet tissue) | | | |
| --- | --- | --- | --- | --- |
| Wild-type females x  wild-type males | Liver  x ± SD | Brain  x ± SD | Small intestine  x ± SD | Kidney  x ± SD |
| Intact mothers  Wild- type males (5) | 30.46 ± 2.37 | 1.38 ± 0,19 | 2.46 ± 0.96 | 1,93 ± 0.22 |
| CuCl_2_ treated mothers  Wild- type males (5) | 45.51 ± 3.19^b^ | 1.32 ± 0.12 | 1.98 ± 0.4 | 1.53 ± 0.38 |
| CuCl_2_-DMDTC treated mothers  Wild- type males (5) | 40.81 ± 5.49^a^ | 1.31 ± 0.25 | 2.20 ± 0.74 | 2.17± 0.19 |
