# Supplementary material for: Prenatal Treatment of Mosaic Mice (Atp7a mo-ms) Mouse Model for Menkes Disease, with Copper Combined by Dimethyldithiocarbamate (DMDTC)
Source: PLoS One. 2012 Jul 18;7(7):e40400. doi: 10.1371/journal.pone.0040400 (PMC3399861; doi:10.1371/journal.pone.0040400)
Supplement: Table S5 — Cu concentration (g/g wet tissue) in the organs of the 14-day-old progeny of the heterozygous mothers. (a) Significantly different from wild-type animals P<0.01; (b) Significantly different from wild-type animals P<0.000; (c) Significantly different from untreated mutant animals P<0.01; (d) Significantly different from untreated mutants P<0.05; (e) Significantly different from untreated mutants P<0.05; (f) Significantly different from wild-type animals P<0.001. (RTF) [file pone.0040400.s006.rtf]

Table S5. 
Parents' genotype	Cu concentration (g/g wet tissue)
	
Heterozygous females x wild-type males	Liver
x  SD	Brain
x  SD	Small intestine
x  SD	Kidney
x  SD	
Untreated mothers, control
Wild-type males (6)
Mutant males (6)	
34.88  8.22
2.30  0.28a
 	
1.42  0.49
0.32  0.15b
	
2,13  0.88
7,06  2,44a
	
1.57  0.31
10,10  1.00a
	
CuCl2 treated mothers
Wild- type males (5)
Mutant males (5)	
47.90  7.06e 
3,71  0,72cf
	
1.29  0.29
0.38  0.21
	
1.93  0.16
12,65  2,42c
	
1.36  0.45
9.96  1,52	
CuCl2-DMDTC treated mothers
Wild-type males (4)
Mutant males (9)
	

29.59  5.88
2.89  0.63	

1.50  0.23
0,46   0.07d
	

1.48  0.22
17.08  1,79c
	

2.08  0.2
7.20  1.60c
	
