# Supplementary material for: Prenatal Treatment of Mosaic Mice (Atp7a mo-ms) Mouse Model for Menkes Disease, with Copper Combined by Dimethyldithiocarbamate (DMDTC)
Source: PLoS One. 2012 Jul 18;7(7):e40400. doi: 10.1371/journal.pone.0040400 (PMC3399861; doi:10.1371/journal.pone.0040400)
Supplement: Table S6 — Cu concentration (g/g wet tissue) in the organs of the wild-type and heterozygous mothers. (RTF) [file pone.0040400.s007.rtf]

Table S6. 

Female genotype /organ	Cu concentration (g/g wet tissue)	
Wild-type	Untreated control (5)	CuCl2-treated (3)	CuCl2-DMDTC- treated (3)	
liver	4.29  0.49	4.60  0.45	3.79  0.20	
kidney	4.22  0.31	4.35  0.23	4.49  0.83	
Heterozygous females				
liver	 3.94  0.31	3.91  0.26	3.49  0.69	
kidney	21.84  7.89	22.06  1.50	22.96  4.74	
